# Avoiding Emergency Surgery With The Use of Sugar in A Patient With Psychotic Disorder and Rectal Prolapse

**DOI:** 10.1192/j.eurpsy.2026.11327

**Published:** 2026-07-31

**Authors:** E. Shaska, A. Panagopoulos, E. Liolis, V.-D. Dafnomili, L. Tchabashvili, K. Tasios, P. Leventis, N. Kornaros, A. Antzoulas, D. Litsas, F. Mulita

**Affiliations:** 1 Head of Department of Emergency Psychiatry, “Ali Mihali” Psychiatric Hospital, Vlora, Albania; 2 Department of Surgery; 3 Department of Oncology, University of Patras, Patras; 4 Department of Surgery, General Hospital of Aigio, Aigio; 5 Department of Surgery, General Hospital of Lamia, Lamia, Greece

## Abstract

**Introduction:**

Though rectal prolapse is uncommon in adolescent patients, there is a noted correlation with rectal prolapse in adult patients who are treated for chronic psychiatric disease.

**Objectives:**

We report a case of a young man with psychosis and rectal prolapse, who was admitted to the emergency department.

**Methods:**

A young man with a psychotic disorder was admitted to the emergency department with perianal pain and a palpable bulging mass, diagnosed with incarcerated rectal prolapse (Figure 1A-D). An initial attempt to manually reduce the prolapse by applying continuous pressure was unsuccessful. Therefore, 20 grams of granulated sugar was applied to the prolapsed tissue for 10 minutes. As the sugar was applied to the mucosa, a reduction of oedema was observed. Eventually, the rectal prolapse was reduced by gentle manual manipulation. The patient was discharged home the following day with advice on dietary changes and gentle laxatives to manage his constipation, which is a risk factor for rectal prolapse.

**Results:**

Rectal prolapse has an annual incidence of 2.5% (per 100000 people); their incidence increases after the fifth decade of life. The condition is more common among women, inmates, and patients with mental disorders. The most common symptoms are constipation, incontinence, incomplete evacuation, rectal bleeding, pain, and tenesmus. Although the spectrum of symptoms varies with the type of rectal prolapse, 50%-75% and 25%-50% of the patients complain of fecal incontinence and constipation, respectively. Patients with internal rectal prolapse of Oxford grades I–III without incontinence, those with internal prolapse of Oxford grade IV with a high surgical risk, and those with minimally symptomatic external prolapse are candidates for conservative therapies. Nonoperative management includes defecation training, use of stool softeners, and dietary changes. Patients should consume 30–40 g of fiber daily and perform at least 100 min of aerobic exercise weekly. Biofeedback therapy, which involves real-time training of pelvic muscle contraction and anal sphincter relaxation in coordination with rectal emptying, may also be beneficial. These treatments do not cure rectal prolapse, but may be useful for improving the quality of life. Surgery should be considered if conservative therapies fail after 2–3 months.

**Conclusions:**

Incarcerated rectal prolapse is a rare pathology and is considered an emergency situation. Using sugar can shift the emergency surgery to an elective one. If left untreated, rectal prolapse may present as an emergency, be it incarceration or strangulation. In an emergency presentation for incarcerated rectal prolapse every attempt should be taken to reduce the prolapsed rectum, if not successful, emergency surgery is indicated, with perineal approach being the first choice

**Disclosure of Interest:**

None Declared

